# Inference of Population Structure of *Leishmania donovani* Strains Isolated from Different Ethiopian Visceral Leishmaniasis Endemic Areas

**DOI:** 10.1371/journal.pntd.0000889

**Published:** 2010-11-16

**Authors:** Tesfaye Gelanew, Katrin Kuhls, Zewdu Hurissa, Teklu Weldegebreal, Workagegnehu Hailu, Aysheshm Kassahun, Tamrat Abebe, Asrat Hailu, Gabriele Schönian

**Affiliations:** 1 Institut für Mikrobiologie und Hygiene, Charité Universitätsmedizin Berlin, Berlin, Germany; 2 Faculty of Medicine, Addis Ababa University, Addis Ababa, Ethiopia; 3 College of Medicine and Health Sciences, University of Gondar, Gondar, Ethiopia; 4 Arba Minch Hospital, Arba Minch, Ethiopia; Lancaster University, United Kingdom

## Abstract

**Background:**

Parasites' evolution in response to parasite-targeted control strategies, such as vaccines and drugs, is known to be influenced by their population genetic structure. The aim of this study was to describe the population structure of Ethiopian strains of *Leishmania donovani* derived from different areas endemic for visceral leishmaniasis (VL) as a prerequisite for the design of effective control strategies against the disease.

**Methodology/Principal Findings:**

Sixty-three strains of *L. donovani* newly isolated from VL cases in the two main Ethiopian foci, in the north Ethiopia (NE) and south Ethiopia (SE) of the country were investigated by using 14 highly polymorphic microsatellite markers. The microsatellite profiles of 60 previously analysed *L. donovani* strains from Sudan, Kenya and India were included for comparison. Multilocus microsatellite typing placed strains from SE and Kenya (n = 30) in one population and strains from NE and Sudan (n = 65) in another. These two East African populations corresponded to the areas of distribution of two different sand fly vectors. In NE and Sudan *Phlebotomus orientalis* has been implicated to transmit the parasites and in SE and Kenya *P. martini*. The genetic differences between parasites from NE and SE are also congruent with some phenotypic differences. Each of these populations was further divided into two subpopulations. Interestingly, in one of the subpopulations of the population NE we observed predominance of strains isolated from HIV-VL co-infected patients and of strains with putative hybrid genotypes. Furthermore, high inbreeding irreconcilable from strict clonal reproduction was found for strains from SE and Kenya indicating a mixed-mating system.

**Conclusions/Significance:**

This study identified a hierarchical population structure of *L. donovani* in East Africa. The existence of two main, genetically and geographically separated, populations could reflect different parasite-vector associations, different ecologies and varying host backgrounds and should be further investigated.

## Author Summary

In the Horn of Africa, visceral leishmaniasis, caused by protozoan parasites of the *Leishmania donovani* complex, continues to pose a major health problem affecting the poorest of the poor. Population genetic studies are crucial for the development of drugs and vaccines against microorganisms. However, our knowledge about the population structure of *L. donovani* parasites in this region is still very limited. Using a highly discriminatory multilocus microsatellite typing approach, we found a remarkably high genetic diversity among the East African strains of *L. donovani* studied which grouped into two genetically and geographically distinct populations comprising parasites from SE and Kenya, and those from NE and Sudan. Despite *Leishmania* being widely regarded as a clonal organism, our results suggest a possible co-existence of clonal and sexually reproducing strains of *L. donovani* from SE. The information obtained by the present study is helpful for future design of parasite-targeted control measures in East Africa.

## Introduction

In Ethiopia, it is estimated that every year more than 4,000 individuals suffer from visceral leishmaniasis (VL, otherwise called kala-azar) caused by protozoan parasites of the *Leishmania donovani* complex [1]. The worst affected region is north Ethiopia (NE) bordering Sudan which accounts for more than 60% of the reported VL cases that are frequently associated with HIV/AIDS [2], [3], [4]. On the other hand, VL is endemic in south Ethiopia (SE) near the border with Kenya, where 20% of cases, rarely associated with HIV/AIDS, occur [4].

VL in the Horn of Africa has important epidemiological and clinical features. For instance, based on the phlebotomine sand fly species involved in the transmission cycle of the parasite, two markedly different ecological situations have been recognized in East African VL foci. One is in the semiarid regions of NE and eastern Sudan [5], [6], [7] where VL is transmitted by *Phlebotomus orientalis* residing in cracks of black cotton clay soils of Acacia-Balanties forest. The second is in the savannah and forest areas in SE and Kenya, where VL is associated with *P. martini* and *P. celiae* thriving in *Macrotermes* termite hills [8], [9]. Studies elsewhere showed that differences in the biology and ecology of sand fly vectors may influence the genetic make-up of the *Leishmania* parasite populations they harbor and transmit [10]. Vectors could possibly select a particular group of parasites or parasites could differ in their adaptability to sand fly species or populations [11]. From this, one could hypothesize that at least two genetically distinct populations of parasites of the *L. donovani* complex should be present in East Africa corresponding to the different vector species. But this remains to be proven by analysing strains of *L. donovani* from the two ecotypes of VL in East Africa. Also, differences in the clinical manifestation of the disease, such as prevalence of post kala-azar dermal leishmaniasis (PKDL) cases, have been documented between the two major East African foci [12], [13]. Moreover, treatment outcomes were recently noted to vary in different VL foci of Ethiopia [14].

The extent to which drug-resistant or virulent parasite strains contribute their genes to the next generations depends on the breeding mode of the organism. Thus understanding the reproductive mode is crucial for the development of drugs and vaccines [15], [16]. Although controversial, the consensus based on the population genetic studies reported so far is that *Leishmania* is a clonal organism [17]. The criteria considered as indicators of clonality in *Leishmania* have, however suffered from the limited discriminatory power of the molecular markers used, e.g. multilocus enzyme electrophoresis (MLEE), random amplification of polymorphic DNA (RAPD) and restriction fragment length polymorphism (RFLP). In addition, linkage disequilibrium, a classical hallmark of clonality, has been recently shown to be an unreliable measure for testifying clonality in an organism [18], [19], [20]. There is also growing evidence of recombination in *Leishmania* at inter-and intra-species level [21], [22], [23], [24], [25]. Indeed, high-resolution markers are necessary to address key epidemiological questions, including the reproductive mode. Microsatellite markers have proved to be the most powerful tools for population genetic studies in *Leishmania* [16], [26], [27]. The power of these markers is basically that they are abundant in the genomes of *Leishmania*, highly informative, neutral, not under selective pressure and co-dominant [27].

Paying attention to the fundamental importance of population genetic studies for unravelling key epidemiological questions associated with VL in Ethiopia in particular, and in East Africa in general, is a prerequisite for the development of effective control strategies. We used a set of highly polymorphic multilocus microsatellite markers [28], [29] to investigate the level of genetic diversity, population structuring and the reproductive system in strains of the *L. donovani* complex strains isolated in different endemic foci in Ethiopia.

## Materials and Methods

### Ethical considerations

This study was conducted in accordance with the Helsinki declaration. It was reviewed and approved by the Institutional Review Board (IRB), Medical Faculty, Addis Ababa University, and the Ethical Committee of Charitè University Medicine. Written informed consent was obtained from each study participant.

### Parasite strains and DNA isolation

**Figure 1:**
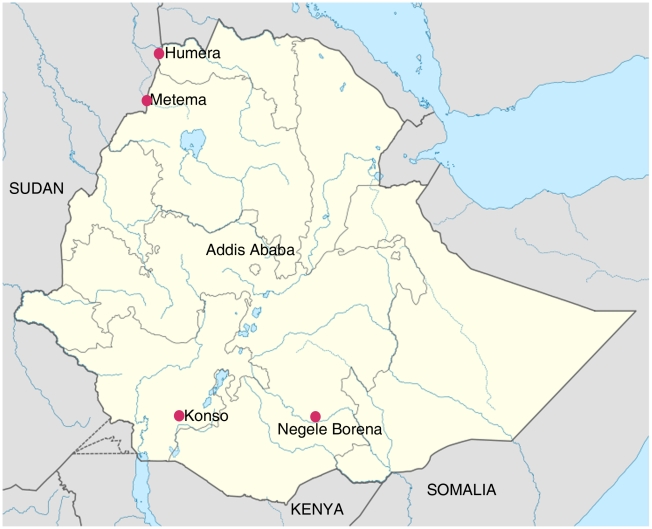
Map of Ethiopia. The VL foci in NE and SE are indicated. The NE foci are bordering the VL foci in Sudan, whereas the SE foci are bordering Kenya.

In this study, we used 63 strains of *L. donovani* isolated between 2007 and 2009 from VL cases in five areas of endemicity in Ethiopia; Metema, Humera and Belessa in NE, and Negele Borena and Konso in SE (Figure 1). Some strains were collected from patients living in different areas non-endemic for VL but having records of travel to one of the VL foci in Ethiopia or Sudan. Additionally, the microsatellite profiles of 60 strains of *L. donovani* previously described by Kuhls et al. [29] and Alam et al. [30] were included for comparison. Three of them were previously isolated in Ethiopia, 21 were from Sudan, 8 from Kenya and 28 from India. Table S1 displays the list of the strains studied, their WHO codes, geographic origins, zymodemes, pathology, and population, subpopulation and cluster assignment.

The Ethiopian strains were cryo-preserved and re-cultured in biphasic Novay-MacNeal-Nicolle (NNN) media. DNA was isolated from pelleted promastigotes using phenol/chlorophorm extraction as previously described [31]. ITS1 polymerase chain reaction-restriction length polymorphism (PCR-RFLP) was carried out to confirm that the 63 newly isolated strains belong to the *L. donovani* complex [32].

### Multilocus microsatellite typing (MLMT)

For the population genetic study, we used 14 unlinked microsatellite markers previously described elsewhere [28], [29]. Fluorescence-labeled forward primers (Proligo, France) were used for the amplification of microsatellite containing sequences applying the PCR conditions described earlier [29]. The precise size of the amplicons was determined on an automated ABI sequencer (SMB Services in Molecular Biology, Berlin Germany), using the peak scanner ABI PRISM GeneMapper software version 3.7 (Applied Biosystems, Foster City, USA) as described earlier [28]. In each run, a reference strain of *L. donovani* (MHOM/IN/80/DD8) was included for which the microsatellite sizes for the 14 loci had been determined by sequencing [29]. MLMtype for each strain was obtained by compiling all alleles at each locus.

### Population genetic analysis

Population structure was examined by a Bayesian model-based clustering approach deployed in STRUCTURE software version 2.1 [33] once the input file was generated with software MSA version 3.0 [34]. This algorithm identifies genetically distinct populations on the basis of allele frequencies and estimates the individuals' membership coefficient in each probabilistic population. The STRUCTURE program was run under a previously described set of parameters [29]. The most probable number of populations (K) was identified by plotting ΔK values of K from 1 to 10 with 10 replicate runs for each K and corresponds to the peak of the ΔK graph [35].

Microsatellite-based genetic distances were calculated based on the proportions of shared alleles distance measure (D_AS_), applying the software POPULATIONS version 1.2.28 (http://bioinformatics.org/~tryphon/populations). Neighbor-Joining (NJ) trees were constructed from the resulting matrix using POPULATIONS and MEGA version 3.1 [36], with bootstrap values (1,000 replicates). Additionally, phylogenetic networks were inferred from the distance matrix obtained from the microsatellite dataset by using the Neighbor-Net method in SplitsTree4 [37]. GDA software was used to quantify allelic richness (A), mean number of alleles (MNA), expected and observed heterozygosity (*H*e and *H*o, respectively) and inbreeding coefficient (*F*_IS_) from the microsatellite data. Pairwise Wright's fixation index (*F*_ST_), which estimates level of genetic differentiation and gene flow between and within populations was calculated using MSA software, assuming microsatellites evolve in accordance with infinite alleles model (IAM).

In addition, BAPS 5 software [38] was used to expose cryptic subpopulations/subclusters within each East African population inferred by STRUCTURE. An input file in Genepop version 3.3 format was used that was generated from microsatellite data using MSA software. For each cluster inferred by BAPS, *F*_IS_ was re-calculated and compared with the *F*_IS_ values for subpopulations identified by STRUCTURE. To see the effect of temporal and geographic origin of strains we divided the Ethiopian *L. donovani* strains by year of isolation and endemic focus and re-calculated the *F*_IS_ values for each subdivision. A significant decrease in *F*_IS_ compared to *F_IS_* values calculated for the subpopulations inferred by STRUCTURE indicates the presence of Wahlund effect due to host, temporal or micro-geographic sub-structuring [16].

## Results

### Multilocus genotypes

Amplification and subsequent estimation of repeat numbers were performed for all 63 Ethiopian strains (n = 22 from SE and n = 41 from NE) over the 14 markers used. Among the 63 strains analysed, 55 distinct MLMTypes were identified. Four strains from SE shared the same MLMType. Four strain pairs also had identical MLMTypes. Two of these pairs, MHOM/ET/2008/Dm62- and MHOM/ET/2008/DM299, and MHOM/ET/2009/DM376sp- and MHOM/ET/2009/DM376SpR, were isolated from the same HIV/VL co-infected patients during different episodes of the disease. The first strain in each pair was isolated before initiation of treatment, and the second during 6-months or one-year follow-ups. The remaining two pairs of genetically identical strains were from different patients, one pair from NE and the other pair from SE.

### Inference of populations and subpopulations

**Figure 2:**
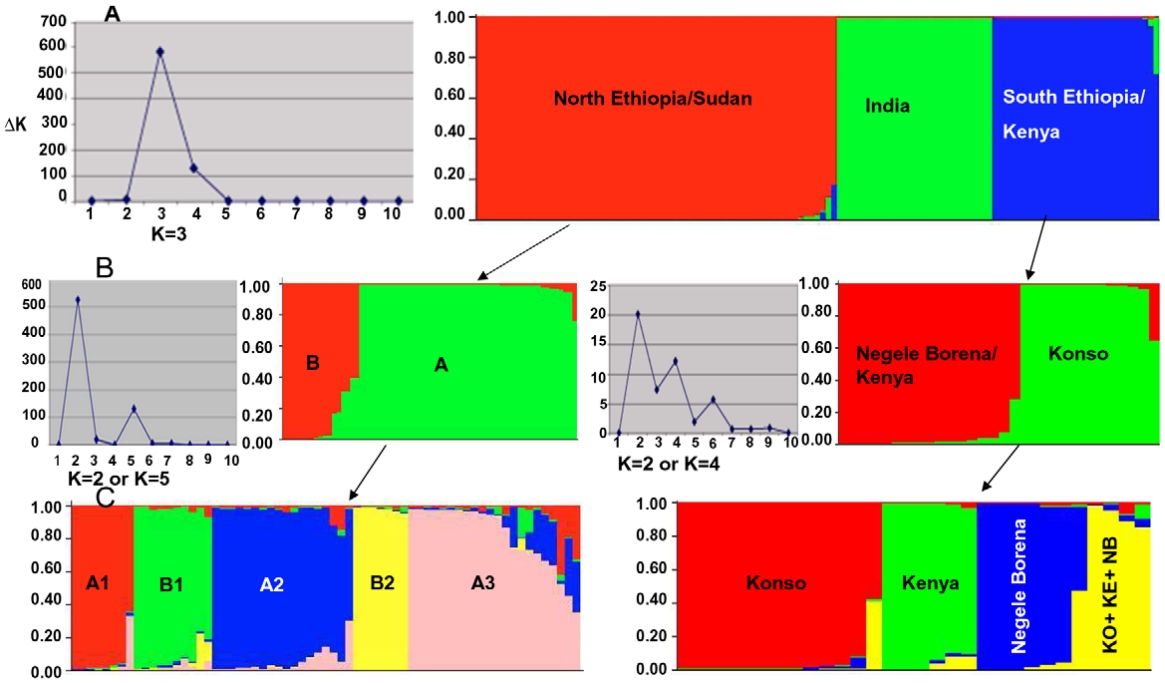
Populations, subpopulations and clusters for 123 *L. donovani* strains as inferred by STRUCTURE. The peak at ΔK represents the most probable number of populations and subpopulations. Presence of smaller peaks in ΔK plot for the subpopulations indicated the presence of further clustering. **A**) Three main populations: North Ethiopia/Sudan (NE/SD), India, and South Ethiopia/Kenya (SE/KE) were identified in the whole data set of 123 strains. **B**) Within each East African population, two subpopulations were identified, A and B in the NE/SD population, and Konso and NB/KE in the SE/KE population. **C**) Five clusters were apparent, three (A1, A2 and A3) in the NE/SD-A subpopulation and two (B1 and B2) in the NE/SD-B subpopulation. Four clusters were detectable in the SE/KE population, named Konso, Kenya, Negele Borena and KO+ KE+NB. Each strain is represented by each vertical line in the STRUCTURE bar plot. Strains with mixed membership to the different populations, subpopulations or clusters are represented by different colored segments of the vertical bar which is proportional to the membership coefficient. The maximum membership coefficient is 1 meaning that a strain is a member in only one population, subpopulation or cluster.

For the inference of population structure and sub-structure, the microsatellite profiles obtained for the 63 strains from Ethiopia were compared to the profiles of 60 previously characterized East African and Indian strains of *L. donovani*. The Bayesian statistic-based algorithm implemented in STRUCTURE assigned the 123 strains, in total, to three distinct populations named onwards as SE/KE, NE/SD, and IND. Population SE/KE contained strains (n = 30) from SE and Kenya, population NE/SD (n = 65) from NE and Sudan, and population IND all strains from India (n = 28) (Table S1 and Figure 2a).

Evanno et al. [35] stated that STRUCTURE accurately detects the uppermost hierarchical level of population structure and that subsequent analyses of subsets defined by the best assignment of individuals to groups provided by the program allow finding the hidden within-group structure. Therefore, to check for sub-structures within the East African populations SE/KE and NE/SD, STRUCTURE analysis was re-run separately for the strains assigned to them. Two major subpopulations were detected in each East African population which were named A and B in case of the NE/SD population, and NB/KE and KO in case of SE/KE population (Figure 2b and Table S1). However, the ΔK peak was weak for the subpopulations KO and NB/KE in SE/KE. All strains belonging to the subpopulation KO (n = 13) were isolated from VL cases in Konso district, whereas the second subpopulation NB/KE comprised all strains from Negele Borena district (n = 4), the 8 strains from Kenya and five strains from Konso. The NE/SD-A subpopulation was further sub-divided into 3 A clusters and 2 B clusters, respectively. Similarly, the South Ethiopian strains split off from the Kenyan strains in further STRUCTURE analyses (Figure 2c and Table S1).

Subpopulation NE/SD-A (n = 48) encompassed 73% of the novel isolates from NE used in this study and 75% of the strains from NE and Sudan analysed in previous studies. Eight of the total sixteen HIV-VL strains clustered in this subpopulation. Of the three clusters found within NE/SD-A, in the cluster A2 (n = 18) all but one strain were old strains mainly from Sudan, whereas clusters A1 and A3 consisted of 9 and 21 news strains from NE, respectively. Interestingly, subpopulation NE/SD-B (n = 17) comprised all old stocks (n = 6) previously identified as one East African population, population 4 [29], plus 11 new strains of which eight were isolated from Ethiopian HIV/VL co-infected patients. The cluster NE/SD-B1 (n = 10) contained strains of putative hybrid or mixed genotypes while the cluster B2 contained strains (n = 7) that could be one of the parents for the putative hybrid/mixed genotypes (Figure 2c). Strains in the NE/SD-B1 cluster showed heterozygosity at 41% of the 14 microsatellite loci. In the six of the loci most of the heterozygous alleles (89%) matched perfectly with homozygote alleles in the two hypothetical parental populations cluster B2 and subpopulation NE/SD-A (Table S2). For instance, the strains MCAN/SD/2000/LEM3946, MHOM/SD/1997/LEM3429, MHOM/SD/1993/GE, MHOM/ET/2007/DM19, MHOM/ET/2007/DM62, MHOM/ET/2008/DM287, MHOM/ET/2008/DM295, MHOM/ET/2008/DM299 and MHOM/ET/2009/DM389, belonging to the cluster NE/SD-B1 displayed two fragments of 78 and 98 bp for locus Li22-35. Almost all strains in subpopulation NE/SD-A were homozygous in this locus presenting the 78 bp allele, whereas seven strains clustering in NE/SD-B2, namely MHOM/ET/2008/DM256, MHOM/ET/2008/DM257, MHOM/ET/2009/DM559, MHOM/ET/2009/DM376S/376R, MHOM/ET/2008/DM481, MHOM/SD/1997/LEM3463 and MHOM/ET/2000/HUSSEN, were homozygous for the 98 bp allele. Interestingly, four of the hybrid strains in NE/SD-B1 were isolated from HIV/VL co-infected AIDS patients.

**Figure 3:**
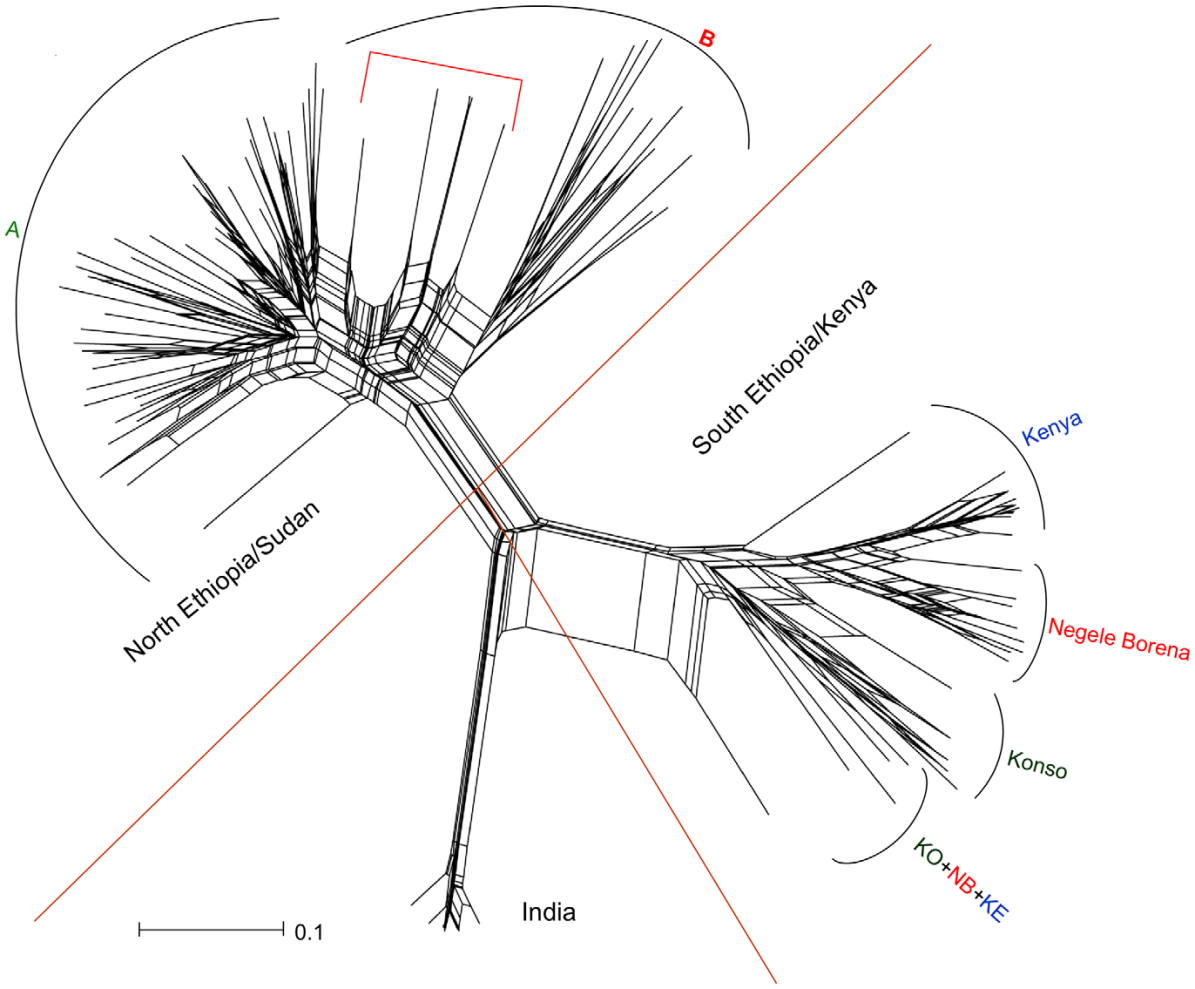
Phylogenetic network (unrooted) for 123 *L. donovani* strains constructed with the Neighbor-Net algorithm. STRUCTURE, NJ tree and network analyses detected the same three main populations as well as the two subpopulations (A and B) in the North Ethiopia/Sudan population and the four clusters; Konso, Negele Borena, Kenya and KO+KE+NB; in the South Ethiopia/Kenya population. The reticulate patterns seen in the network indicates either hybridization or recombination events between similar or closely related genotypes. The putative hybrid genotypes are indicated by a red bar line on the top. They are located in between their hypothetical parental strains in NE/SD-A subpopulation A and in cluster B1 of the NE/SD subpopulation B.

The three populations (NE/SD, SE/KE and IND) and the division of the NE/SD population into two main subpopulations and further into 5 clusters, as inferred by STRUCTURE, was congruent with the topologies of the *D*_AS_-NJ tree (data not shown) and of the SplitsTree (Figure 3). The two subpopulations of NE/SD as well as the four groups, KO, NB, KE and NB-KE, in population SE/KE were also depicted by the NJ analyses. Furthermore, the reticulate patterns seen in the SplitsTree between and within the three main populations with putative hybrid strains in intermediate positions indicate the possible occurrence of hybridization or recombination events.

### Characterization of the main East African populations

The genetic diversity in each population was measured by determining the number of alleles per locus (allelic richness, A), expected (*H*e) and observed (*H*o) heterozygosities as well the inbreeding coefficient (*F*_IS_). As shown in Table S3, there is considerable polymorphism in all loci but not all of them were polymorphic within the individual populations. Three loci [Li46-67(C), Li71-5/2(P) and Li71-5/2(Q)] were entirely monomorphic within population SE/KE but different between SE/KE and NE/SD, and four to nine different alleles were seen in population NE/SD for these markers. Locus Lm2TG was entirely monomorphic in population NE/SD but different between NE/SD and SE/KE. The number of alleles per locus ranged from one to 10 (mean 5.5) for NE/SD, and one to 15 (mean 4.1) for SE/KE. The most polymorphic locus for SE/KE population was locus CS20 with 15 alleles, whereas for NE/SD it was locus Li23-41(F) with 11 alleles (Table S3). Population-specific alleles were found for the loci Lm2TG, Li22-35(E), Li23-41(F), Li45-24(G), CS20 and KLIST7031 by which strains belonging SE/KE could be unambiguously distinguished from strains belonging NE/SD. This implies that one could track the spread of *L. donovani* parasites using only these markers in East Africa.

The expected heterozygosity (*H*e) which is a measure for genetic diversity was slightly higher in strains of *L. donovani* from NE/SD (*He* = 0.47) as compared to strains from SE/KE (*He* = 0.39). It cannot be excluded that this might be due to the fact that more strains from NE (N = 44; 41 new plus 3 old) and Sudan (n = 21) were analysed compared to the 30 strains from SE (n = 22) and Kenya (n = 8).

In both East African populations identified herein, a considerable deficit of heterozygotes (*H*e > *H*o, *F*_IS_ >0) was observed for both single-locus and multilocus data (Table S3). To determine whether heterozygote deficiency could be attributed to population sub-structuring, the Wahlund effect, the *F*_IS_ values were re-calculated for each subpopulation and cluster defined by STRUCTURE (Table S4). This resulted in only a slight reduction of *F*_IS_ values for the subpopulations NE/SD-A and –B as well as for subpopulations SE/KE-KO and –NB/KE. However, the *F*_IS_ values were much higher for the two subpopulations in SE/KE than for the two subpopulations in NE/SD. The *F*_IS_ values remained significantly positive when re-calculated for the 5 and 4 clusters in NE/SD and SE/KE, respectively, except for the cluster NE/SD-B1 (Table S4).

We also used BAPS to check for the presence of further hidden subclustering within each East African population and found 10 (with probability of *P*_BAPS_ = 0.57) and 7 (*P*_BAPS_ = 0.91) clusters in SE/KE and NE/SD, respectively. For each cluster identified by BAPS, except for those containing single strains only, the *F*_IS_ values were re-calculated. For NE/SD, the results showed a significant decrease of *F*_IS_ in BAPS clusters with a mean of 0.22 (0.07–0.40) (Table S5). However, the *F*_IS_ values for the BAPS' clusters in SE/KE remained clearly positive ranging from 0.34 to 0.86 (Table S5) and indicating the presence of significant inbreeding in this population.

Further, we analysed Ethiopian strains of *L. donovani* alone defined by year of isolation and geographic origin. First, we calculated *F*_IS_ values for the NE (n = 41) and SE (n = 22) populations separately. These values *F*_IS_ = 0.42 for NE and *F*_IS_ = 0.83 for SE, were similar to those obtained when Ethiopian and Sudanese (n = 65) as well as Ethiopian and Kenyan strain (n = 30) were taken together (Table S4). When the strains from SE were divided into two groups, SE-2007 and SE-2008, and strains from NE into three groups, NE-2007, NE-2008 and NE-2009, according to the year of their isolation, the *F*_IS_ for each division remained clearly positive. For the SE groups it ranged from 0.67 to 0.85, and for the NE groups from 0.29 to 0.49. We also re-calculated *F*_IS_ for SE strains isolated in the two foci, Konso and Nebele Borena within two years (NB-2007, Konso-2007 and Konso-2008), and found high positive values (Table S4).We did not re-calculate *F*_IS_ values for the NE strains isolated in the same year in same focus. Because most of our patients were soldiers and immigrant workers and had to travel frequently the two foci Humera and Metema, it was impossible to accurately record where they got infected.

### F-statistics

The genetic differentiation between populations and subpopulations as inferred by STRUCTURE was tested by F-statistics. The estimated pairwise *F*_ST_ values between populations and all but one subpopulations ranged from 0.312 to 0.727 (p = 0.0001) (Table 1). This indicated that the two main populations NE/SD and SE/KE and the subpopulations in NE/SD are clearly genetically isolated with only limited gene flow between them. The two subpopulations in SE/KE, KO and NB/KE, however showed less genetic differentiation. We also looked for genetic differentiation between the subpopulations of only Ethiopian *L. donovani* defined by geographic origin and year of isolation; SE-2007, SE-2008 SE-2007-Konso, SE-2008-Konso, SE-2008-NB for the SE strains, and NE-2007, NE-2008 and NE-2009 for the NE strains. Strong genetic differentiation (*F*_ST_ = 0.56; *p*<.0001) was only found between NE and SE similar to what was obtained for NE/SD and SE/KE. The *F*_ST_ values between the different subdivisions were not significant (data not shown).

**Table 1:** *F*_ST_ estimates with corresponding p values for pair-wise comparisons of the populations and subpopulations of *L. donovani* identified by STRUCTURE analysis.

Populations/subpopulations	*F*_ST_	p
**SE/KE vs. NE/SD**	0.494	0.0001
**SE/KE vs. IND**	0.727	0.0001
**NE/SD vs. IND**	0.597	0.0001
**KO vs. NB/KE**	0.041	0.0006
**A vs. B**	0.312	0.0001

### Discussion

Highly discriminatory methods differentiating the parasites at the strain level are needed to answer key epidemiological and clinical questions associated with VL in East Africa. The MLMT approach has been shown to combine high discriminatory power with good reproducibility allowing the comparison of profiles identified in different laboratories and at different times [26], [27]. Therefore, a panel of 14 microsatellite markers was used in the present study for elucidating the genetic diversity, the population structure and the reproductive system of *L. donovani* in Ethiopia and for comparing the microsatellite profiles found with those of previously analyzed strains from Sudan and Kenya as well as from India. To the best of our knowledge, this study is the first one that has investigated a large number of strains in the *L. donovani* complex from different areas of endemicity in Ethiopia using the MLMT approach. Only three Ethiopian strains, all from north-western foci, were previously typed by using MLMT [29].

Using different types of population genetic analysis, namely Bayesian statistics- and distance-based models as well as *F*-statistics, we exposed two main populations among the 95 East African strains of *L. donovani* which were clearly separate from the population comprising strains from India. The two East African populations, one consisting of strains from northern Ethiopia and Sudan, NE/SD, and the other of strains from southern Ethiopia and Kenya, SE/KE, represented genetically isolated populations and were both further divided in two subpopulations. The existence of two genetically distinct populations in Ethiopia is not surprising considering the fact that the Ethiopian foci of VL are adjacent to well-known foci, either in Sudan or Kenya [39], [40] and that the two foci, NE and SE, are very distant (Figure 1). Because the NE foci are geographically adjacent to Sudanese foci of VL, and the same is true for SE and Kenyan foci, people have always moved across these borders for trading or seeking employment in big commercial farms or due to ongoing conflicts with high military activities [41].

The presence of two geographically and genetically isolated populations of *L. donovani* in East Africa is supported by differences in clinical behavior and biology of the strains from the two foci. In NE, as well as in Sudan, the transmission of VL is suggested to be anthropozoonotic, at least occasionally [42], [43]. In SE and Kenya, VL is an anthroponotic disease. Also, PKDL is relatively uncommon in SE and Kenya and develops several years after apparent cure of VL patients, whereas in 56% of the Sudanese patients lesions appear within weeks or months [12]. In NE, cases of PKDL are increasing with 13% occurring in non-HIV positive and 27% in HIV-coinfected individuals [13]. Interestingly, the two main populations identified in this study correlate with the areas of distribution of the two different phlebotomine sand fly species that transmit parasites of the *L. donovani* complex in Ethiopia: *P. orientalis* in NE, as in Sudan, and *P. martini* in SE, as in Kenya. It is certainly worthwhile investigating whether the population genetic structure of *L. donovani* in East Africa may be influenced by the existence of these different species of sand fly vectors. However, we cannot rule out that other factors such as population migration routes, ecological variations, varying host backgrounds etc., or a combination of these and other factors might be responsible for the observed genetic differentiation.

The two major subpopulations as well as three of the four clusters within SE/KE correlated to the geographical origins of the strains studied. Most strains from Konso grouped in subpopulation SE/KE-KO and were clearly differentiated from strains from Negele Borena and from Kenyan strains. However, few strains from Konso were assigned to the clusters comprising strains from Negele Borena and Kenya which might reflect intertribal movement since Negele Borena is situated between Kenya and Konso. The results could be, however biased by the small number of strains per cluster and should be verified by analyzing more strains from different SE and Kenyan foci. On the contrary, no correlation could be found between the geographical origin of strains and the two subpopulations and the 5 clusters in population NE/SD. This is in agreement with a recent MLMT-based population genetic study on Sudanese strains of *L. donovani* [44] and consistent with high rate of population movement between these Sudanese and Ethiopian foci [45]. Instead, the subpopulations and clusters identified in NE/SD might reflect the differences in hosts (population–specific positive selection), reservoirs and the biology of the vectors [10], [46], and year of isolation. Subpopulation NE/SD-A, further divided into clusters A1 to A3, comprised most of the newly isolated NE strains (A1 and A3) and old Sudanese strains (A2). Subpopulation NE/SD-B, further divided into B1 and B2, on the other hand, comprised NE strains isolated from HIV-positive VL patients and old strains from NE and Sudan for which the immunological status of their hosts is unknown. Only three newly isolated strains from HIV-negative patients were found in subpopulation NE/SD-B. The predominance of strains from HIV/VL co-infected individuals in NE/SD-B compared to NE/SD-A may indicate that these strains are less virulent and hence may cause only asymptomatic infections in immune-competent hosts. Also, clonal spread of hybrid genotypes after recombination event(s), as suggested by the low *F*_IS_ obtained for the cluster B1, could explain the predominance of strains from HIV co-infected cases in subpopulation NE/SD-B.

The distinction between true relapse due to recrudescence of original parasites after treatment, and re-infection by a new parasite is important for the accurate estimation of anti-*Leishmania* drug efficacy. This is of particular importance for treatment follow-up and drug trial studies in Ethiopia where the rate of HIV/VL co-infection and associated relapses are on rise [2], [3], [13], [47]. In the present study, pairs of strains were collected from two HIV/VL co-infected patients; one strain before the start of treatment and the other during follow-up. MLMT showed that the strains from first and subsequent episodes were genetically identical suggesting a true relapse and thus treatment failure in these two patients. Taking together the result of the present study and previous findings [48], [49], MLMT appears to be an useful tool for differentiating true relapses from re-infections.

*L. donovani* has been considered to be a clonal diploid organism [17] in which *F*_IS_ values are supposed to be negative due to heterozygote deficiency [50]. Contrary to this, we found high inbreeding coefficients within all but one East African clusters which could result from population subdivision (Wahlund effect), presence of null alleles, natural selection, genetic conversion and inbreeding as discussed by Rougeron et al. [16]. In our study, all strains were amplified at all microsatellite loci. Thus, the high *F*_IS_ values that were observed across all polymorphic loci are unlikely to be due to the presence of null alleles. Selection may cause underdominance by decreasing the fitness of heterozygous genotypes and gene conversion could lead to a transition from the heterozygous to the homozygous stage [16]. In both cases, varying *F*_IS_ should be expected across our 14 non-coding microsatellite loci. In our general dataset, little variance of *F*_IS_ values was observed across these loci (Table S3). This variance is even decreasing when only the Ethiopian parasite populations and subpopulations were analysed (Table S3 and Table S4).

To expose hidden subpopulation structure we used the BAPS software. For the BAPS clusters of the population NE/SD the *F*_IS_ values were lower than those estimated for the NE/SD subpopulations and clusters inferred by STRUCTURE, indicating that the latter were at least partly due to population subdivision (Wahlund effect). There was however, no indication for temporal clustering as we still found high inbreeding for NE strains isolated in the same year but with no significant differentiation between different years of isolation. For the BAPS clusters and subclusters of the population SE/KE as well as for the SE strains alone subdivided into different clusters based on the same year isolation at the same focus, the *F*_IS_ values remained highly positive suggesting that the Wahlund effect is not responsible for the high inbreeding observed. Thus our results are incompatible with the clonal hypothesis of *Leishmania* and provide evidence for high inbreeding or mating between closely related genotypes, especially among the strains from SE and Kenya but also, to a lower extent, for the strains from NE and Sudan. Similar finding i.e., extreme inbreeding, indicating mixed-mating events was observed in *L. braziliensis* [16]. The circumstantial evidence of “strong clonality” in *Leishmania* might be spurious since most of the previous studies were based on the less polymorphic genetic markers and used the insensitive linkage disequilibrium as a clonal index. Whether reproductive strategies of strains from SE are different from those of strains from NE is not clear at the moment. It deserves further investigations which should involve more strains for both populations that are not separated by time and geography.

We found relatively high level of heterozygosity across the various microsatellite loci in strains from NE/SD when compared to strains from SE/KE which is in agreement with a recent study in Sudan [44]. This could be explained by mutation in one of the double alleles [51] or by a recombination event among different genotypes in *L. donovani*. Mutation in one of the double alleles would be the most likely explanation for the relatively higher amount of heterozygote loci in NE/SD which is characteristic for clonal diploid organisms. However, the presence of homozygote alleles, one in subpopulation NE/SD-A and the other in cluster NE/SD-B2, which perfectly match to the heterozygote alleles in NE/SD-B1, indicate that the heterozygous allele combinations might have arisen from recombination between different genotypes rather than from a simple mutation [52]. The results of our STRUCTURE and phylogenetic network analyses are in favor of this hypothesis.

Natural hybrids on inter- as well as intra-species level have been repeatedly reported [21], [22], [23], [24], [25]. Six putative hybrid strains were identified in the present study. Interestingly, some of the putative hybrid strains grouping in NE/SD-B1 were isolated from HIV/AIDS patients. Previously, two strains representing *L. infantum*/*L. major* hybrids, were isolated from immuno-compromised patients in Portugal [24] and it was hypothesized that immuno-compromised patients could be infected with more than one genotype over long periods of time, with large numbers of amastigotes which could provide opportunities for the hybridization between different *Leishmania* genotypes. Although recombination in *Leishmania* was proven to occur rather in the invertebrate hosts than mammalian hosts [53], it is reasonable to link the recovery of putative hybrid strains in HIV co-infected patients with the clonal selection of hybrid genotypes. Hybrid strains may have a selective advantage over homozygote strains, e.g. by enhancing the transmission potential [54]. It would be interesting to clone primary cultures of strains showing mixed membership in different populations, subpopulations or clusters, and to re-analyze them by MLMT in order to find out whether they might represent true natural hybrids.

In conclusion, the present study revealed the presence of remarkable genetic heterogeneity among East African strains of *L. donovani*. East African *L. donovani* parasites are not only genetically distinct from the Indian but the parasites from Kenya and south Ethiopia in one hand and those from Sudan and northwest Ethiopia in the other are also distinct. This study also sheds some light on understanding of the population structure and reproductive pattern of East African *L. donovani*. This information, together with future epidemiological and population genetic studies will be very useful for the design of parasite-targeted control strategies which aim to eradicate VL in East Africa.

## Supporting Information

Table S1 Designation and characteristics of *Leishmania donovani* strains used in this study.
(0.23 MB DOC)

Table S2 Microsatellite profiles of putative hybrid strains and their corresponding hypothetical parents for the six microsatellite loci.
(0.04 MB DOC)

Table S3 Descriptive statistics: polymorphism, heterozygosity and inbreeding at the 14 microsatellite loci compared for the East African populations (SE/KE and NE/SD) as inferred by STRUCTURE and for the Ethiopian populations alone (SE and NE).
(0.13 MB DOC)

Table S4 Comparison of FIS values between populations, sub-populations and clusters defined by geographical origin and year of isolation for Ethiopian strains of *L. donovani* alone (n = 63), and populations, subpopulations and clusters identified for all *L. donovani* strains from East Africa analysed in this study (n = 95).
(0.06 MB DOC)

Table S5 Descriptive statistics: polymorphism, heterozygosity and inbreeding for the BAPS clusters.
(0.04 MB DOC)
