# Supplementary material for: Inference of Population Structure of Leishmania donovani Strains Isolated from Different Ethiopian Visceral Leishmaniasis Endemic Areas
Source: PLoS Negl Trop Dis. 2010 Nov 16;4(11):e889. doi: 10.1371/journal.pntd.0000889 (PMC2982834; doi:10.1371/journal.pntd.0000889)
Supplement: Table S1 — Designation and characteristics of Leishmania donovani strains used in this study. (0.23 MB DOC) [file pntd.0000889.s001.doc]

Table S1

Designation and characteristics *of Leishmania donovani* strains used in this study

| **WHO code** | **Country** | **District-Travel history** | **MON** | **Pathology** | **Population** | **Subpopulation** | **Cluster** | **Source** |
| --- | --- | --- | --- | --- | --- | --- | --- | --- |
| MHOM/ET/2007/DM3 | Ethiopia | Konso | nd | VL | SE/KE | KO | KO | this study |
| MHOM/ET/2007/DM9 | Ethiopia | Konso | nd | VL | SE/KE | KO | KO | this study |
| MHOM/ET/2007/DM15 | Ethiopia | Konso | nd | VL | SE/KE | KO | KO | this study |
| MHOM/ET/2007/DM16 | Ethiopia | Konso | nd | VL | SE/KE | KO | KO | this study |
| MHOM/ET/2007/DM18 | Ethiopia | Konso | nd | VL | SE/KE | KO | KO | this study |
| MHOM/ET/2007/DM63 | Ethiopia | Konso | nd | VL | SE/KE | KO | KO | this study |
| MHOM/ET/2007/DM65 | Ethiopia | Negele Borena | nd | VL | SE/KE | NB/KE | NB | this study |
| MHOM/ET/2007/DM76 | Ethiopia | Konso | nd | VL | SE/KE | KO | NB | this study |
| MHOM/ET/2008/DM261 | Ethiopia | Konso | nd | VL | SE/KE | NB/KE | NB | this study |
| MHOM/ET/2008/DM280 | Ethiopia | Konso | nd | VL | SE/KE | NB/KE | KO | this study |
| MHOM/ET/2008/DM281 | Ethiopia | Konso | nd | VL | SE/KE | KO | KO | this study |
| MHOM/ET/2008/DM282 | Ethiopia | Konso | nd | VL | SE/KE | KO | KO | this study |
| MHOM/ET/2008/DM283 | Ethiopia | Konso | nd | VL | SE/KE | NB/KE | NB | this study |
| MHOM/ET/2008/DM284 | Ethiopia | Negele Borena | nd | VL | SE/KE | NB/KE | NB | this study |
| MHOM/ET/2008/DM285 | Ethiopia | Konso | nd | VL | SE/KE | KO | KO+KE+NB | this study |
| MHOM/ET/2008/DM288 | Ethiopia | Konso | nd | VL | SE/KE | NB/KE | KO+KE+NB | this study |
| MHOM/ET/2008/DM289 | Ethiopia | Konso | nd | VL | SE/KE | KO | KO | this study |
| MHOM/ET/2008/DM290 | Ethiopia | Konso | nd | VL | SE/KE | KO | KO | this study |
| MHOM/ET/2008/DM291 | Ethiopia | Negele Borena | nd | VL | SE/KE | NB/KE | NB | this study |
| MHOM/ET/2008/DM292 | Ethiopia | Negele Borena | nd | VL | SE/KE | NB/KE | NB | this study |
| MHOM/ET/2008/DM311 | Ethiopia | Konso | nd | VL | SE/KE | KO | KO | this study |
| MHOM/ET/2009/DM317 | Ethiopia | Konso | nd | VL | SE/KE | KO | KO | this study |
| MHOM/KE/1983/NLB189 | Kenya | nd | 37 | PKDL | SE/KE | NB/KE | KE | KIT |
| MHOM/KE/1984/NLB218 | Kenya | nd | nd | PKDL | SE/KE | NB/KE | KO+KE+NB | KIT |
| MHOM/KE/1985/NLB323 | Kenya | nd | 37 | VL | SE/KE | NB/KE | KE | KIT |
| MHOM/KE/??/LRC-L445 | Kenya | nd | nd | nd | SE/KE | NB/KE | KE | LRC |
| MHOM/KE/1954/LRC-L53 | Kenya | nd | 36 | nd | SE/KE | NB/KE | KE | LRC |
| MHOM/KE/1973/MRC74 | Kenya | nd | 2 | nd | SE/KE | NB/KE | KO+KE+NB | LSTHM |
| IMAR/KE/1962/L57 | Kenya | nd | 37 | nd | SE/KE | NB/KE | KE | LSTHM |
| MHOM/KE/1955/LRC-L53 | Kenya | nd | 36 | nd | SE/KE | NB/KE | KE | LRC |
| MHOM/ET/2007/DM4 | Ethiopia | nd | nd | VL | NE/SD | A | A3 | this study |
| MHOM/ET/2007/DM5 | Ethiopia | Gondar | nd | VL | NE/SD | A | A3 | this study |
| MHOM/ET/2007/DM6 | Ethiopia | Gondar | nd | VL | NE/SD | A | A3 | this study |
| MHOM/ET20/2007/DM7 | Ethiopia | Gondar | nd | VL | NE/SD | A | A1 | this study |
| MHOM/ET/2007/DM11 | Ethiopia | Gondar | nd | VL | NE/SD | A | A3 | this study |
| MHOM/ET/2007/DM14 | Ethiopia | Gonder | nd | VL | NE/SD | A | A1 | this study |
| MHOM/ET/2007/DM19 | Ethiopia | Gondar | nd | VL | NE/SD | B | B1 | this study |
| MHOM/ET/2007/DM20 | Ethiopia | Gondar | nd | VL | NE/SD | A | A1 | this study |
| MHOM/ET/2007/DM26 | Ethiopia | Gonder | nd | VL | NE/SD | A | A3 | this study |
| MHOM/ET/2007/DM62^a^ | Ethiopia | Libo Kemkem-Abdurafi | nd | VL/HIV+ | NE/SD | B | B1 | this study |
| MHOM/ET/2007/DM64 | Ethiopia | Gonder | nd | VL | NE/SD | A | A1 | this study |
| MHOM/ET/2007/DM111 | Ethiopia | Gonder | nd | VL/HIV+ | NE/SD | A | A3 | this study |
| MHOM/ET/2007/DM139 | Ethiopia | Gonder | nd | VL/HI+ | NE/SD | A | A3 | this study |
| MHOM/ET/2008/DM254 | Ethiopia | Belessa | nd | VL | NE/SD | A | A1 | this study |
| MHOM/ET/2008/DM255 | Ethiopia | Military-suspected NE endemic | nd | VL/HIV+ | NE/SD | A | A3 | this study |
| MHOM/ET/2008/DM256 | Ethiopia | Military-suspected NE endemic | nd | VL/HIV+ | NE/SD | B | B2 | this study |
| MHOM/ET/2008/DM257 | Ethiopia | Gondar-Humera | nd | VL/HIV+ | NE/SD | B | B2 | this study |
| MHOM/ET/2008/DM258 | Ethiopia | Gondar | nd | VL | NE/SD | A | A3 | this study |
| MHOM/ET/2008/DM259 | Ethiopia | Bihar Dar-Humera | nd | VL | NE/SD | A | A3 | this study |
| MHOM/ET/2008/DM260 | Ethiopia | N/Gondar | nd | VL | NE/SD | A | A3 | this study |
| MHOM/ET/2008/DM272 | Ethiopia | N/Gondar | nd | VL | NE/SD | A | A3 | this study |
| MHOM/ET/2008/DM273 | Ethiopia | W/Gojam-Humera | nd | VL/HIV+ | NE/SD | A | A3 | this study |
| MHOM/ET/2008/DM275 | Ethiopia | Gojam-Humera | nd | VL | NE/SD | A | A3 | this study |
| MHOM/ET/2008/DM276 | Ethiopia | N/Gondar-Humera | nd | VL | NE/SD | A | A1 | this study |
| MHOM/ET/2008/DM277 | Ethiopia | Ambagiorgis, N/Gondar | nd | VL | NE/SD | A | A1 | this study |
| MHOM/ET/2008/DM278 | Ethiopia | Gondar | nd | VL | NE/SD | A | A3 | this study |
| MHOM/ET/2008/DM286 | Ethiopia | Enfraz, N/Gondar | nd | VL | NE/SD | A | A1 | this study |
| MHOM/ET/2008/DM287 | Ethiopia | W/Gojam-East Sudan | nd | VL | NE/SD | B | B1 | this study |
| MHOM/ET/2008/DM294 | Ethiopia | Wolega-Metema | nd | VL | NE/SD | A | A3 | this study |
| MHOM/ET/2008/DM295 | Ethiopia | Belessa | nd | VL | NE/SD | B | B1 | this study |
| MHOM/ET/2008/DM296 | Ethiopia | Gondar | nd | VL/HIV+ | NE/SD | A | A3 | this study |
| MHOM/ET/2008/DM297 | Ethiopia | Debub Wollo | nd | VL | NE/SD | A | A2 | this study |
| MHOM/ET/2008/DM299^a^ | Ethiopia | Libo Kemkem-Abdurafi relapse of DM-62 | nd | VL/HIV+ | NE/SD | B | B1 | this study |
| MHOM/ET/2009/DM376sp^b^ | Ethiopia | Addis Ababa-travel history | nd | VL/HIV+ | NE/SD | B | B2 | this study |
| MHOM/ET/2009/DM376spR^b^ | Ethiopia | Addis Ababa-Relapse | nd | VL/HIV+ | NE/SD | B | B2 | this study |
| MHOM/ET/2009/DM389 | Ethiopia | Gondar | nd | VL/HIV+ | NE/SD | B | B1 | this study |
| MHOM/ET/2009/DM446 | Ethiopia | Gondar | nd | VL/HIV+ | NE/SD | A | A3 | this study |
| MHOM/ET/2009/DM451 | Ethiopia | Gondar | nd | VL/HIV+ | NE/SD | A | A1 | this study |
| MHOM/ET/2009/DM483 | Ethiopia | Addis Ababa-travel history | nd | VL/HIV+ | NE/SD | A | A3 | this study |
| MHOM/ET/2009/DM559 | Ethiopia | Gondar | nd | VL-Uk HIV status | NE/SD | B | B2 | this study |
| MHOM/ET/2009/DM607 | Ethiopia | N/Gondar Armachio | nd | VL | NE/SD | A | A3 | this study |
| MHOM/SD/1975/LV139 | Sudan | nd | nd | CL | NE/SD | A | A3 | KIT |
| MHOM/SD/1962/LRC-L61 | Sudan | nd | nd | nd | NE/SD | A | A2 | KIT |
| MHOM/SD/1968/IS | Sudan | nd | nd | VL | NE/SD | A | A2 | KIT |
| MHOM/SD/1992/51-band | Sudan | nd |  |  | NE/SD | A | A2 | KIT |
| MHOM/SD/1993/GE | Sudan | Gedaref | 82 | VL | NE/SD | B | B1 | KIT |
| MHOM/SD/1993/AEB | Sudan | nd | 82 | VL | NE/SD | A | A2 | KIT |
| MHOM/SD/1993/45-UMK | Sudan | nd | 30 | VL | NE/SD | A | A2 | KIT |
| MHOM/SD/1993/762L | Sudan | nd | 30 | VL | NE/SD | A | A2 | KIT |
| MHOM/SD/1993/9S | Sudan | Gedaref | 18 | VL | NE/SD | A | A2 | KIT |
| MHOM/SD/1993/452BM | Sudan | nd | 30 | PKDL | NE/SD | A | A2 | KIT |
| MHOM/SD/1993/597LN | Sudan | nd | ? | ? | NE/SD | A | A2 | KIT |
| MHOM/SD/1993/38-UMK | Sudan | nd | ? | ? | NE/SD | A | A2 | KIT |
| MHOM/SD/1993/35-band | Sudan | Gedaref | 82 | VL | NE/SD | A | A2 | KIT |
| MHOM/SD/1993/597-2 | Sudan | nd | 30 | PKDL | NE/SD | A | A2 | KIT |
| MHOM/SD/1993/338 | Sudan | nd | 18 | PKDL | NE/SD | A | A2 | KIT |
| MHOM/ET/1967/HU3 | Ethiopia | Humera | 18 | VL | NE/SD | A | A2 | LRC |
| MHOM/ET/2000/HUSSEN | Ethiopia | nd | 83/31 | VL | NE/SD | B | B1 | LSTHM |
| MHOM/SD/1982/GILANI | Sudan | nd | 30 | VL | NE/SD | A | A2 | LSTHM |
| MHOM/ET/1972/GEBRE1 | Ethiopia | nd | 82 | VL | NE/SD | A | A2 | LSTHM |
| MHOM/SD/1997/LEM3429 | Sudan | Gedaref | 82 | VL | NE/SD | B | B1 | LEMPP |
| MHOM/SD/1997/LEM3463 | Sudan | Gedaref | 82 | VL | NE/SD | B | B2 | LEMPP |
| MCAN/SD/2000/LEM3946 | Sudan | nd | 274 | VL | NE/SD | B | B1 | LEMPP |
| MHOM/SD/1962/3S | Sudan | nd | 81 | VL | NE/SD | A | A2 | LEMPP |
| MHOM/SD/1997/LEM3472 | Sudan | nd | 267 | PKDL | NE/SD | B | B1 | LEMPP |
| MHOM/IN/1998/DD8 | India | nd | 2 | VL | IND | IND | IND | LEMPP |
| MHOM/IN/2000/DEVI | India | Bihar | 2 | VL | IND | IND | IND | LSTHM |
| MHOM/IN/1996/THAK35 | India | Bihar | 2 | nd | IND | IND | IND | LSTHM |
| MHOM/IN/2001/BHU20140 | India | Bihar | nd | VL | IND | IND | IND | BHU |
| MHOM/IN/2002/BHU1 | India | Bihar | nd | VL | IND | IND | IND | BHU |
| MHOM/IN/2002/BHU2 | India | Bihar | nd | VL | IND | IND | IND | BHU |
| MHOM/IN/2002/BHU3 | India | Bihar | nd | VL | IND | IND | IND | BHU |
| MHOM/IN/2002/BHU4 | India | Bihar | nd | VL | IND | IND | IND | BHU |
| MHOM/IN/2002/BHU5 | India | Bihar | nd | VL | IND | IND | IND | BHU |
| MHOM/IN/2002/BHU6 | India | Bihar | nd | VL | IND | IND | IND | BHU |
| MHOM/IN/2002/BHU7 | India | Bihar | nd | VL | IND | IND | IND | BHU |
| MHOM/IN/2002/BHU8 | India | Bihar | nd | VL | IND | IND | IND | BHU |
| MHOM/IN/2002/BHU9 | India | Bihar | nd | VL | IND | IND | IND | BHU |
| MHOM/IN/2002/BHU11 | India | Bihar | nd | VL | IND | IND | IND | BHU |
| MHOM/IN/2002/BHU12 | India | Bihar | nd | VL | IND | IND | IND | BHU |
| MHOM/IN/2002/BHU13 | India | Bihar | nd | VL | IND | IND | IND | BHU |
| MHOM/IN/2002/BHU15 | India | Bihar | nd | VL | IND | IND | IND | BHU |
| MHOM/IN/2002/BHU17 | India | Bihar | nd | VL | IND | IND | IND | BHU |
| MHOM/IN/2002/BHU20 | India | Bihar | nd | VL | IND | IND | IND | BHU |
| MHOM/IND/03/BHU50 | India | Bihar | nd | VL | IND | IND | IND | BHU |
| MHOM/IND/03/BHU53 | India | Bihar | nd | VL | IND | IND | IND | BHU |
| MHOM/IND/03/BHU55 | India | Bihar | nd | VL | IND | IND | IND | BHU |
| MHOM/IND/03/BHU37 | India | Bihar | nd | VL | IND | IND | IND | BHU |
| MHOM/IND/03/BHU41 | India | Bihar | nd | VL | IND | IND | IND | BHU |
| MHOM/IND/03/BHU32 | India | Bihar | nd | VL | IND | IND | IND | BHU |
| MHOM/IND/03/BHU52 | India | Bihar | nd | VL | IND | IND | IND | BHU |
| MHOM/IND/03/BHU54 | India | Bihar | nd | VL | IND | IND | IND | BHU |
| MHOM/IND/03/BHU33 | India | Bihar | nd | VL | IND | IND | IND | BHU |

Nd, not determined: VL, visceral leishmaniasis; PKDL, post kala-azar dermal leishmaniasis,

Population, subpopulation and cluster assignment as inferred by STRUCTURE: population NE/SD contained all strains from northwest Ethiopia and Sudan, population SE/KE contained all strains from south Ethiopia and Kenya and Population IND contained all strains from India. Strains with ^a^, ^b^ were isolated from the same patients at different episodes of VL.

KIT: Royal Tropical Institute Amsterdam, The Netherlands (H. Schallig); LRC: Leishmania Reference Centre, Hebrew University-Hadassah Medical School, Jerusalem, Israel (L.F. Schnur); LSTHM: London School of Hygiene and Tropical Medicine, UK (I. Mauricio); LEMPP: Centre National de Reference des Leishmaniosis, Montpellier, France (J.P. Dedet, P. Bastien); BHU: Banares Hindu University, Varanasi, India (S. Sundar).
