# Supplementary material for: Inference of Population Structure of Leishmania donovani Strains Isolated from Different Ethiopian Visceral Leishmaniasis Endemic Areas
Source: PLoS Negl Trop Dis. 2010 Nov 16;4(11):e889. doi: 10.1371/journal.pntd.0000889 (PMC2982834; doi:10.1371/journal.pntd.0000889)
Supplement: Table S2 — Microsatellite profiles of putative hybrid strains and their corresponding hypothetical parents for the six microsatellite loci. (0.04 MB DOC) [file pntd.0000889.s002.doc]

Table S2

Microsatellite profiles of putative hybrid strains and their corresponding hypothetical parents for the six microsatellite loci

|  | **Li41-56(B)** | **Li45-67(C)** | **Li22-35(E)** | **Li71-33(P)** | **Li71-7(R)** | **CS20** |
| --- | --- | --- | --- | --- | --- | --- |
| **Pa** | **92** | **74** | **78** | **89** | **92** | **91-95^a^** |
| **Pb** | **94** | **76** | **98** | **103** | **94** | **87** |
| MHOM/ET/2007/DM19 | 92/92 | 74/76 | 78/98 | 89/103 | 94/94 | 91/87 |
| MHOM/ET/2007/DM62 | 92/94 | 74/76 | 78/98 | 89/103 | 92/94 | 93/95 |
| MHOM/ET/2008/DM287 | 92/94 | 74/76 | 78/98 | 89/103 | 92/94 | 93/95 |
| MHOM/ET/2008/DM295 | 92/94 | 74/76 | 78/98 | 89/103 | 92/94 | 89/95 |
| MHOM/ET/2008/DM299 | 92/94 | 74/76 | 78/98 | 89/103 | 92/94 | 93/95 |
| MHOM/ET/2009/DM389 | 92/94 | 74/76 | 78/98 | 89/103 | 92/94 | 91/95 |
| MCAN/SD/2000/LEM3946^b^ | 86/94 | 76/76 | 78/98 | 117/103 | 94/94 | 95/87 |
| MHOM/SD/1997/LEM3429 | 92/94 | 76/76 | 78/98 | 117/103 | 94/94 | 95/95 |
| MHOM/SD/1993/GE | 92/94 | 76/76 | 78/98 | 117/103 | 94/94 | 91/95 |
| MHOM/ET/2000/Hussen | 92/92 | 74/76 | 98/98 | 117/103 | 94/94 | 87/87 |

Pa, hypothetical parental strain in NE/SD-A subpopulation; Pb, hypothetical parental strain in NE/SD B subpopulation cluster B2; strains with WHO code are putative hybrids in the NE/SD-B subpopulation B cluster B1. Most of the hypothetical parental strains are homozygote for these loci. The numbers given in the table refer to the allele size (in bp) of the selected microsatellite loci.

^a^ the parental strains displayed variable allele size between 91 and 95 bp for locus CS20

^b^MCAN/SD/2000/LEM3946 was reported to be a putative hybrid by multilocus sequence typing [52].
