# Supplementary material for: Inference of Population Structure of Leishmania donovani Strains Isolated from Different Ethiopian Visceral Leishmaniasis Endemic Areas
Source: PLoS Negl Trop Dis. 2010 Nov 16;4(11):e889. doi: 10.1371/journal.pntd.0000889 (PMC2982834; doi:10.1371/journal.pntd.0000889)
Supplement: Table S3 — Descriptive statistics: polymorphism, heterozygosity and inbreeding at the 14 microsatellite loci compared for the East African populations (SE/KE and NE/SD) as inferred by STRUCTURE and for the Ethiopian populations alone (SE and NE). (0.13 MB DOC) [file pntd.0000889.s003.doc]

Table S3

Descriptive statistics: polymorphism, heterozygosity and inbreeding at the 14 microsatellite loci compared for the East African populations (SE/KE & NE/SD) as inferred by STRUCTURE and for the Ethiopian populations alone (SE & NE)

| **Microsatellite Marker** | **Population** | **N** | **A** | ***H*e** | ***H*o** | ***F*_IS_** |
| --- | --- | --- | --- | --- | --- | --- |
| **Lm2TG** | SE/KE | 30 | 5 | 0.636 | 0.010 | 0.845 |
|  | SE | 22 | 4 | 0.586 | 0.091 | 0.849 |
|  | **NE/SD** | **65** | **1** | **na** | **na** | **na** |
|  | **NE** | **41** | **1** | **na** | **na** | **na** |
| **TubCA** | SE/KE | 30 | 6 | 0.730 | 0.01 | 0.865 |
|  | SE | 22 | 4 | 0.555 | 0.091 | 0.840 |
|  | NE/SD | 65 | 2 | 0.030 | 0.0 | 1.000 |
|  | NE | 41 | 2 | 0.048 | 0.000 | 1.000 |
| **Lm4TA** | SE/KE | 30 | 3 | 0.314 | 0.033 | 0.895 |
|  | SE | 20 | 2 | 0.089 | 0.000 | 1.000 |
|  | NE/SD | 65 | 7 | 0.691 | 0.261 | 0.623 |
|  | NE | 41 | 7 | 0.699 | 0.269 | 0.620 |
| **Li41-56(B)** | SE/KE | 30 | 5 | 0.594 | 0.033 | 0.944 |
|  | SE | 22 | 3 | 0.449 | 0.045 | 0.901 |
|  | NE/SD | 65 | 6 | 0.616 | 0.507 | 0.178 |
|  | NE | 41 | 6 | 0.600 | 0.366 | 0.394 |
| **Li46-67(C)** | **SE/KE** | **30** | **1** | **na** | **na** | **na** |
|  | **SE** | **22** | **1** | **na** | **na** | **na** |
|  | NE/SD | 65 | 4 | 0.517 | 0.369 | 0.288 |
|  | NE | 41 | 4 | 0.531 | 0.366 | 0.314 |
| **Li22-35(E)** | SE/KE | 30 | 3 | 0.594 | 0.066 | 0.889 |
|  | SE | 22 | 2 | 0.426 | 0.045 | 0.895 |
|  | NE/SD | 65 | 4 | 0.367 | 0.153 | 0.583 |
|  | NE | 41 | 2 | 0.347 | 0.146 | 0.581 |
| **Li23-41(F)** | SE/KE | 30 | 5 | 0.690 | 0.068 | 0.901 |
|  | SE | 22 | 4 | 0.624 | 0.048 | 0.925 |
|  | NE/SD | 65 | 10 | 0.772 | 0.323 | 0.583 |
|  | NE | 41 | 7 | 0.747 | 0.366 | 0.513 |
| **Li45-24(G)** | SE/KE | 30 | 3 | 0.413 | 0.000 | 1.000 |
|  | SE | 20 | 2 | 0.169 | 0.000 | 1.000 |
|  | NE/SD | 65 | 4 | 0.324 | 0.136 | 0.520 |
|  | NE | 41 | 3 | 0.162 | 0.122 | 0.248 |
| **Li71-33(P)** | **SE/KE** | **30** | **1** | **na** | **na** | **na** |
|  | **SE** | **22** | **1** | **na** | **na** | **na** |
|  | NE/SD | 65 | 9 | 0.388 | 0.246 | 0.368 |
|  | NE | 41 | 8 | 0.386 | 0.244 | 0.371 |
| **Li71-5/2(Q)** | **SE/KE** | **30** | **1** | **na** | **na** | **na** |
|  | **SE** | **22** | **1** | **na** | **na** | **na** |
|  | NE/SD | 65 | 4 | 0.540 | 0.353 | 0.347 |
|  | NE | 41 | 3 | 0.415 | 0.293 | 0.298 |
| **Li71-7(R)** | SE/KE | 30 | 5 | 0.545 | 0.010 | 0.819 |
|  | SE | 22 | 5 | 0.293 | 0.136 | 0.540 |
|  | NE/SD | 65 | 2 | 0.353 | 0.140 | 0.603 |
|  | NE | 41 | 2 | 0.339 | 0.225 | 0.339 |
| **CS20** | SE/KE | 30 | 15 | 0.825 | 0.266 | 0.680 |
|  | SE | 22 | 11 | 0.725 | 0.273 | 0.698 |
|  | NE/SD | 65 | 8 | 0.629 | 0.292 | 0.537 |
|  | NE | 41 | 8 | 0.562 | 0.171 | 0.699 |
| **KLIST7031** | SE/KE | 30 | 2 | 0.033 | 0.033 | 0.000 |
|  | SE | 22 | 2 | 0.045 | 0.045 | 0.000 |
|  | NE/SD | 65 | 9 | 0.737 | 0.646 | 0.125 |
|  | NE | 41 | 8 | 0.703 | 0.732 | -0.041 |
| **KLIST7039** | SE/KE | 30 | 2 | 0.065 | 0.000 | 1.000 |
|  | SE | 22 | 2 | 0.089 | 0.000 | 1.000 |
|  | NE/SD | 65 | 5 | 0.638 | 0.343 | 0.463 |
|  | NE | 41 | 5 | 0.619 | 0.325 | 0.478 |
| **Mean** | SE/KE | 30 | 4.07 | 0.388 | 0.057 | 0.854 |
|  | SE | 22 | 3.2 | 0.289 | 0.055 | 0.812 |
|  | NE/SD | 65 | 5.42 | 0.472 | 0.270 | 0.428 |
|  | NE | 41 | 4.7 | 0.440 | 0.259 | 0.415 |

N, number of strains; A, allelic richness per locus; *He*, expected heterozygosity; *Ho* observed heterozygosity; *F_IS_,* inbreeding coefficient or deviation from panmixia; SE, South Ethiopia; NE; North Ethiopia; SE/KE, South Ethiopia/Kenya; NE/SD, North Ethiopia/Sudan, na, not applicable because the microsatellite locus was monomorphic for all strains in the population.
