# Supplementary material for: Inference of Population Structure of Leishmania donovani Strains Isolated from Different Ethiopian Visceral Leishmaniasis Endemic Areas
Source: PLoS Negl Trop Dis. 2010 Nov 16;4(11):e889. doi: 10.1371/journal.pntd.0000889 (PMC2982834; doi:10.1371/journal.pntd.0000889)
Supplement: Table S4 — Comparison of FIS values between populations, sub-populations and clusters defined by geographical origin and year of isolation for Ethiopian strains of L. donovani alone (n = 63), and populations, subpopulations and clusters identified for all L. donovani strains from East Africa analysed in this study (n = 95). (0.06 MB DOC) [file pntd.0000889.s004.doc]

Table S4.

Comparison of *F*_IS_ values between populations, sub-populations and clusters defined by geographical origin and year of isolation for Ethiopian strains of *L. donovani* alone (n=63), and populations, subpopulations and clusters identified for all *L. donovani* strains from East Africa analysed in this study (n=95)

| **Population** | **Subpopulation** | **Cluster** | **N** | ***H*_e_** | ***H*_o_** | ***F*_IS_** |
| --- | --- | --- | --- | --- | --- | --- |
| **East African *L. donovani* strains as whole** | | | | | | |
| NE/SD  (n=65, *F*_IS_=0.428) | A  (*F*_IS_=0.325) | A1 | 8 | 0.135 | 0.107 | 0.218 |
|  |  | A2 | 18 | 0.358 | 0.289 | 0.196 |
|  |  | A3 | 22 | 0.332 | 0.266 | 0.203 |
|  | B  (*F*_IS_=0.334) | B1 | 10 | 0.464 | 0.435 | 0.064 |
|  |  | B2 | 7 | 0.353 | 0.183 | 0.499 |
| SE/SD  (n=30, *F*_IS_=0.854) | KO  (*F*_IS_=0.730) | KO | 13 | 0.100 | 0.027 | 0.730 |
|  | NB/KE  (*F*_IS_=0.814) | KE | 6 | 0.262 | 0.083 | 0.733 |
|  |  | NB | 7 | 0.196 | 0.091 | 0.551 |
|  |  | NB +KE | 4 | 0.431 | 0.053 | 0.891 |
| **New Ethiopian *L. donovani* strains only** | | | | | | |
| NE  (n=41, *F*_IS_=0.415) | NE-2007 |  | 13 | 0.352 | 0.254 | 0.286 |
|  | NE-2008 |  | 20 | 0.438 | 0.248 | 0.441 |
|  | NE-2009 |  | 8 | 0.538 | 0.295 | 0.470 |
| SE  (N=22, *F*_IS_=0.803) | SE-2007 |  | 8 | 0.211 | 0.071 | 0.676 |
|  | SE-2008 |  | 14 | 0.330 | 0.051 | 0.850 |
|  |  | SE-2007-KO | 7 | 0.170 | 0.041 | 0.774 |
|  |  | SE-2007-NB | 4 | 0.230 | 0.143 | 0.410 |
|  |  | SE-2008-KO | 12 | 0.319 | 0.054 | 0.838 |

n= strains number, *H*_e_= expected heterozygosity, *H*_o_=observed heterozygosity, *F*_IS_, inbreeding coefficient or deviation from panmixia; NE, North Ethiopia; SE, South Ethiopia; KO, Konso; NB, Negele Borena, KE, Kenya; SE/KE, South Ethiopia/Kenya; NE/SD, North Ethiopia/Sudan
