# Supplementary material for: Inference of Population Structure of Leishmania donovani Strains Isolated from Different Ethiopian Visceral Leishmaniasis Endemic Areas
Source: PLoS Negl Trop Dis. 2010 Nov 16;4(11):e889. doi: 10.1371/journal.pntd.0000889 (PMC2982834; doi:10.1371/journal.pntd.0000889)
Supplement: Table S5 — Descriptive statistics: polymorphism, heterozygosity and inbreeding for the BAPS clusters. (0.04 MB DOC) [file pntd.0000889.s005.doc]

Table S5

Descriptive statistics: polymorphism, heterozygosity and inbreeding for the BAPS clusters

| **BAPS cluster** | **N** | ***H*_e_** | ***H*_o_** | ***F*_IS_** |
| --- | --- | --- | --- | --- |
| **NE/SD Population (7 BAPS clusters)** | | | | |
| A1 | 7 | 0.122 | 0.102 | 0.178 |
| A2-a | 19 | 0.350 | 0.312 | 0.112 |
| A2-b | 3 | 0.219 | 0.142 | 0.400 |
| A3 | 19 | 0.311 | 0.255 | 0.183 |
| B1 | 10 | 0.464 | 0.435 | 0.064 |
| B2-a | 6 | 0.301 | 0.190 | 0.389 |
| B2-b | 1 | nd | nd | nd |
| **SE/KE Population (10 BAPS clusters)** | | | | |
| KO-1 | 12 | 0.074 | 0.024 | 0.863 |
| NB-1 | 4 | 0.206 | 0.142 | 0.342 |
| NB-2 | 4 | 0.155 | 0.071 | 0.578 |
| KE-1 | 3 | 0.114 | 0.047 | 0.636 |
| KE-2 | 2 | 0.178 | 0.178 | 0.000 |
| KE-3 | 1 | nd | nd | nd |
| KE-4 | 1 | nd | nd | nd |
| NB-3 | 1 | nd | nd | nd |
| NB-4 | 1 | nd | nd | nd |
| KO-2 | 1 | nd | nd | nd |

**N** = strains number, *H*_e_= expected heterozygosity, *H*_o_=observed heterozygosity, *F*_IS_= inbreeding coefficient or deviation from panmixia, nd= not determined
